# Down-Regulation of Glucose-Regulated Protein (GRP) 78 Potentiates Cytotoxic Effect of Celecoxib in Human Urothelial Carcinoma Cells

**DOI:** 10.1371/journal.pone.0033615

**Published:** 2012-03-16

**Authors:** Kuo-How Huang, Kuan-Lin Kuo, Shyh-Chyan Chen, Te-I Weng, Yuan-Ting Chuang, Yu-Chieh Tsai, Yeong-Shiau Pu, Chih-Kang Chiang, Shing-Hwa Liu

**Affiliations:** 1 Graduate Institute of Toxicology, College of Medicine, National Taiwan University, and National Taiwan University Hospital, Taipei, Taiwan; 2 Department of Urology, College of Medicine, National Taiwan University, and National Taiwan University Hospital, Taipei, Taiwan; 3 Department of Forensic Medicine, College of Medicine, National Taiwan University, and National Taiwan University Hospital, Taipei, Taiwan; 4 Department of Oncology, College of Medicine, National Taiwan University, and National Taiwan University Hospital, Taipei, Taiwan; 5 Department of Integrated Diagnostics and Therapeutics, College of Medicine, National Taiwan University, and National Taiwan University Hospital, Taipei, Taiwan; Wayne State University School of Medicine, United States of America

## Abstract

Celecoxib is a selective cyclooxygenase-2 (COX-2) inhibitor that has been reported to elicit anti-proliferative response in various tumors. In this study, we aim to investigate the antitumor effect of celecoxib on urothelial carcinoma (UC) cells and the role endoplasmic reticulum (ER) stress plays in celecoxib-induced cytotoxicity. The cytotoxic effects were measured by MTT assay and flow cytometry. The cell cycle progression and ER stress-associated molecules were examined by Western blot and flow cytometry. Moreover, the cytotoxic effects of celecoxib combined with glucose-regulated protein (GRP) 78 knockdown (siRNA), (−)-epigallocatechin gallate (EGCG) or MG132 were assessed. We demonstrated that celecoxib markedly reduces the cell viability and causes apoptosis in human UC cells through cell cycle G1 arrest. Celecoxib possessed the ability to activate ER stress-related chaperones (IRE-1α and GRP78), caspase-4, and CCAAT/enhancer binding protein homologous protein (CHOP), which were involved in UC cell apoptosis. Down-regulation of GRP78 by siRNA, co-treatment with EGCG (a GRP78 inhibitor) or with MG132 (a proteasome inhibitor) could enhance celecoxib-induced apoptosis. We concluded that celecoxib induces cell cycle G1 arrest, ER stress, and eventually apoptosis in human UC cells. The down-regulation of ER chaperone GRP78 by siRNA, EGCG, or proteosome inhibitor potentiated the cytotoxicity of celecoxib in UC cells. These findings provide a new treatment strategy against UC.

## Introduction

Bladder urothelial carcinoma (UC) ranks fourth in incidence among cancers in men and eighth in women in the United States [1]. The prognosis for patients with metastatic UC remains poor [2]. Even with chemotherapeutic treatment, the overall median survival is about one year [2]. Cisplatin-based chemotherapy is the standard treatment of patients with metastatic UC; however, despite regimens such as the cisplatin, gemcitabine or paclitaxel combination, the overall response rates vary between 40% and 65% [3]–[5]. The other limiting factor associated with current chemotherapeutic regimens is the substantial toxicities. Therefore, there is an urgent need for the development of novel therapeutic agents for UC treatment.

Celecoxib is a selective inhibitor of cyclooxygenase-2 (COX-2) and is widely used for anti-inflammation or pain control. Considerable preclinical evidence supports the potential of celecoxib against several types of malignancies [6]; however, the utility of celecoxib by itself or in combination with other therapies for treating UC has not been fully explored [7]–[10]. Several studies have reported that celecoxib possesses the anti-tumor effect in the absence of COX-2 involvement [8], [11]. The previous studies have shown that anti-tumor mechanisms of celecoxib may include the death receptors, mitochondria-mediated pathways, cell cycle arrest, Akt phosphorylation inhibition, endoplasmic reticulum (ER) stress, and autophagy [8], [11]–[17]. The exact underlying mechanisms of the anti-tumor effects mediated by celecoxib remain unclear.

The unfolded protein response (UPR) is a cellular stress response of the ER. The ER stress response is activated in response to an accumulation of unfolded or misfolded proteins in the lumen of the ER [18]–[20]. These unfolded proteins can be removed by ER-associated degradation (ERAD), which delivers abnormal proteins to the proteasomes [19], [21]–[22]. In this study, we try to investigate the role of UPR in celecoxib-induced cytotoxicity in human bladder UC cells. We also examine whether the interference of UPR pathway can enhance the celecoxib-induced cytotoxicity in UC cells.

## Materials and Methods

### Cell culture

We have performed the experiments on three cell lines. SV-HUC cells were the SV40 transformed immortalized, non-tumorigenic human urothelial cell line [23]. NTUB1 cells were derived at National Taiwan University Hospital from the surgical specimen of a 70-year-old female patient with high grade transitional cell carcinoma and was proved to be tumorigenic in nude mice [24]–[25]. The T24 cells were derived from a highly malignant grade III human urinary bladder carcinoma (Figure S1). NTUB1 cells were kindly provided from Dr. Yeong-Shiau Pu (Department of Urology, National Taiwan University Hospital, Taipei, Taiwan). T24 human UC cell line was obtained from the American Type Culture Collection (Manassas, VA). SV-HUC cells were kindly provided from Dr. Tai-Lung Cha (Department of Urology, Tri-Service General Hospital and National Defense Medical Center, Taipei, Taiwan). Cells were maintained at 37°C in RPMI-1640 medium (for NTUB1 cells), Dulbecco's Modified Eagle Medium (for T24 cells) or F12 (for SV-HUC) supplemented with 10% fetal bovine serum (FBS), 100 U/mL penicillin, and 100 µg/mL streptomycin (Invitrogen, Carlsbad, CA).

### Reagents and antibodies

Celecoxib pure compound was provided by Pfizer (New York, NY). Various concentrations of celecoxib were prepared as suspensions in DMSO (Sigma-Aldrich, St. Louis, MO) and then mixed with cell medium containing 10% FBS. LM-1685, a celecoxib analogue, is another COX-2 inhibitor purchased from Calbiochem (San Diego, USA). (−)-Epigallocatechin gallate (EGCG) and MG132 (a proteasome inhibitor) were purchased from Sigma-Aldrich (San Diego, USA). Antibodies against various proteins for Western blot analysis such as poly (ADP-ribose) polymerase (PARP), cleaved PARP, caspase-3, 4, 7, 8, 9, cleaved caspase-3, 7, 8, 9, p21, p27, IRE-1α, GRP78, CHOP, and calnexin were obtained from Cell Signaling Technologies (Danvers, MA). Other antibodies against ubiquitin, β-actin and α-tubulin were purchased from Santa Cruz Biotechnology (Santa Cruz, CA), and anti-GAPDH antibody was purchased from Genetex (Irvine, CA).

### Measurement of cell viability

Cell viability was determined by using 3-(4,5-dimethylthiazol-2-yl)- 2,5-diphenyl tetrazolium (MTT, Sigma-Aldrich). In brief, cells were seeded with culture medium in 96-well microplates (4500 cells/well) and incubated at 37°C for 24 h before drug exposures. At the end of treatments with drugs, cells were incubated with completed-medium containing 0.4 mg/ml MTT at 37°C for 4 h. The reduced MTT crystals were dissolved in DMSO and the absorbance was detected at 570 nm with a plate reader.

### Immunoblotting

After various treatments, the NTUB1 and T24 cells were washed with cold phosphate buffered saline (PBS) and then lysed with cell lysis buffer (Cell Signaling Technologies) on ice. The cell lysates were centrifuged at 14000 rpm for 30 min at 4°C. The supernatants were collected and the concentrations of the proteins were determined by BCA protein assay (Thermo Scientific Pierce, Rockford, IL). Equal quantity of each samples were resolved in SDS-polyacrylamide then transfer to polyvinylidene fluoride (PVDF) membrane (Millipore, Billerica, MA). The membranes were incubated with 5% skim milk in PBS and then incubate appreciate amount of primary antibodies in PBS at 4°C overnight. The membranes were then washed twice with PBST (PBS containing 0.05% Tween 20) and incubated at room temperature for 1 h with applicable horseradish peroxidase (HRP)-conjugated secondary antibodies (Genetex) at appropriate dilution ratios in PBS. After washed twist with PBST, antibody bound-membranes were visualized by enhanced chemiluminescence western blotting detection reagents (Millipore).

### Knockdown of GRP78 by using siRNA

For knockdown of GRP78, the cells were transfected with small interfering RNA (siRNA) against GRP78. NTUB1 and T24 cells were transfected with various concentrations of siRNA for GRP78 (Thermo Scientific Dharmacon, Lafayette, CO) or nonsilencing scramble siRNA with the use of SiLenFect (Bio-Red) according to the manufacturer's instructions. The transfected cells were incubated with or without 100 µM celecoxib in complete medium for 24 h.

### Analysis of apoptosis by fluorescence-activated cell sorting (FACS)

Cells were stained with annexin V-FITC apoptosis detection kit (Strong Biotech, Taipei, Taiwan), and apoptotic cells identified and quantified by flow cytometry. Briefly, after exposing to different treatments, NTUB1 and T24 cells were washed with PBS and then harvested by trypsin-EDTA solution (Invitrogen). The cell suspensions were centrifuged at 1000 rpm for 5 min to remove trypsin-EDTA solution. Then the cells were re-suspended and incubated with propidium iodide (PI), annexin V-FITC, and annexin V binding buffer for 15 min at room temperature. The stained cells were analyzed on a FACS flow cytometry (Becton Dickinson Cockeysville, MD).

### Cell cycle analysis by flow cytometry

NTUB1 and T24 cells were grown in medium as mentioned above. At 50% confluency, cells were treated with DMSO control or 100 µM celecoxib for 24 h. Cells were collected and processed for cell cycle analysis. Briefly, 0.5×10^5^ cells were suspended in 0.5 mL of PI solution, and incubated 30 min in the dark. Cell cycle distribution was then analyzed by FACS flow cytometry (Becton Dickinson).

### Statistical analysis

The GraphPad Prism® 4 software was used to perform all data analysis. All data were expressed as mean ± SD and analyzed by one-way ANOVA followed by Bonferroni post hoc test, with values of P<0.05 considered statically significant.

## Results

### Celecoxib affected viability, apoptosis, GRP78 protein expression, and cell cycle in human UC cells

**Figure 1:**
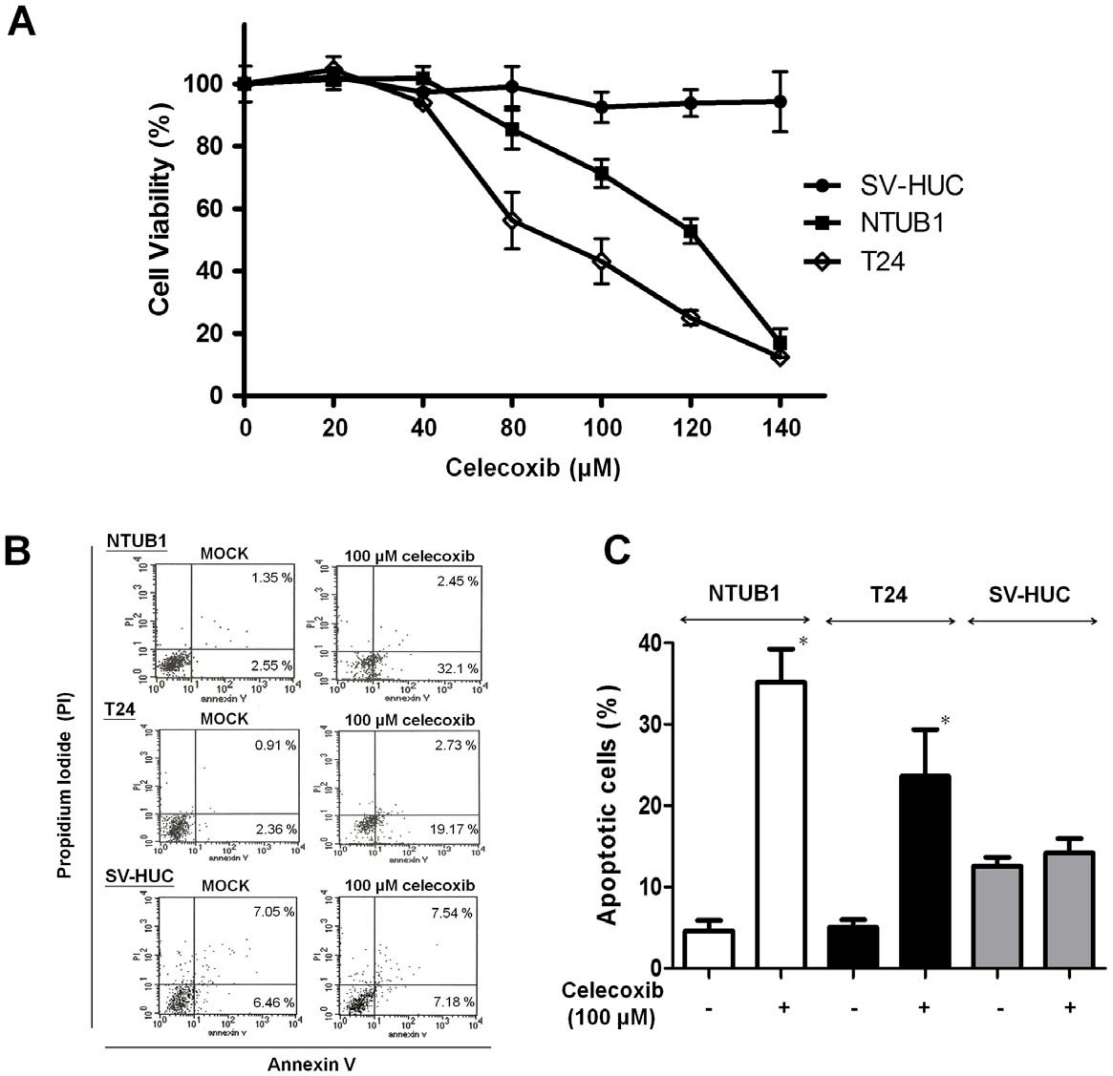
Celecoxib reduces cell viability and induces apoptosis in UC cells and SV-HUC cells. (A) Cells were treated with various concentrations of celecoxib for 24 h. Cell viability was assessed by MTT assay. (B) Cells were exposed to mock (untreated) and 100 µM celecoxib for 24 h. Apoptotic cells were analyzed by FACS flow cytometry with propidium iodide (PI) and annexin V-FITC staining. The lower-right panel presented annexin V-positive cells (early apoptotic cells); the upper-right panel presented late apoptotic cells with membranes permeable to PI and annexin V staining. (C) Quantitative analysis of total apoptosis (early and late) population following 100 µM celecoxib treatment was presented. In (A) and (C), data are presented as means ± SD of three independents experiments. * p<0.05 as compared with control. In (B), results shown are representative of at least three independent experiments.

We first assessed the effect of celecoxib on the viability of human UC cell lines (NTUB1 and T24) and SV-HUC cells using the MTT assay. After 24 h exposure, celecoxib effectively reduced cell viability in a dose-dependent manner in NTUB1 and T24 cells and had no significant effect on cell viability of SV-HUC (Figure 1A). Moreover, apoptotic cells were analyzed by flow cytometry (FACS) with propidium iodide (PI) and Annexin V-FITC staining. Celecoxib (100 µM) markedly induced the cell apoptosis in NTUB1 and T24 cells after 24 h exposure (Figures 1B and C).

**Figure 2:**
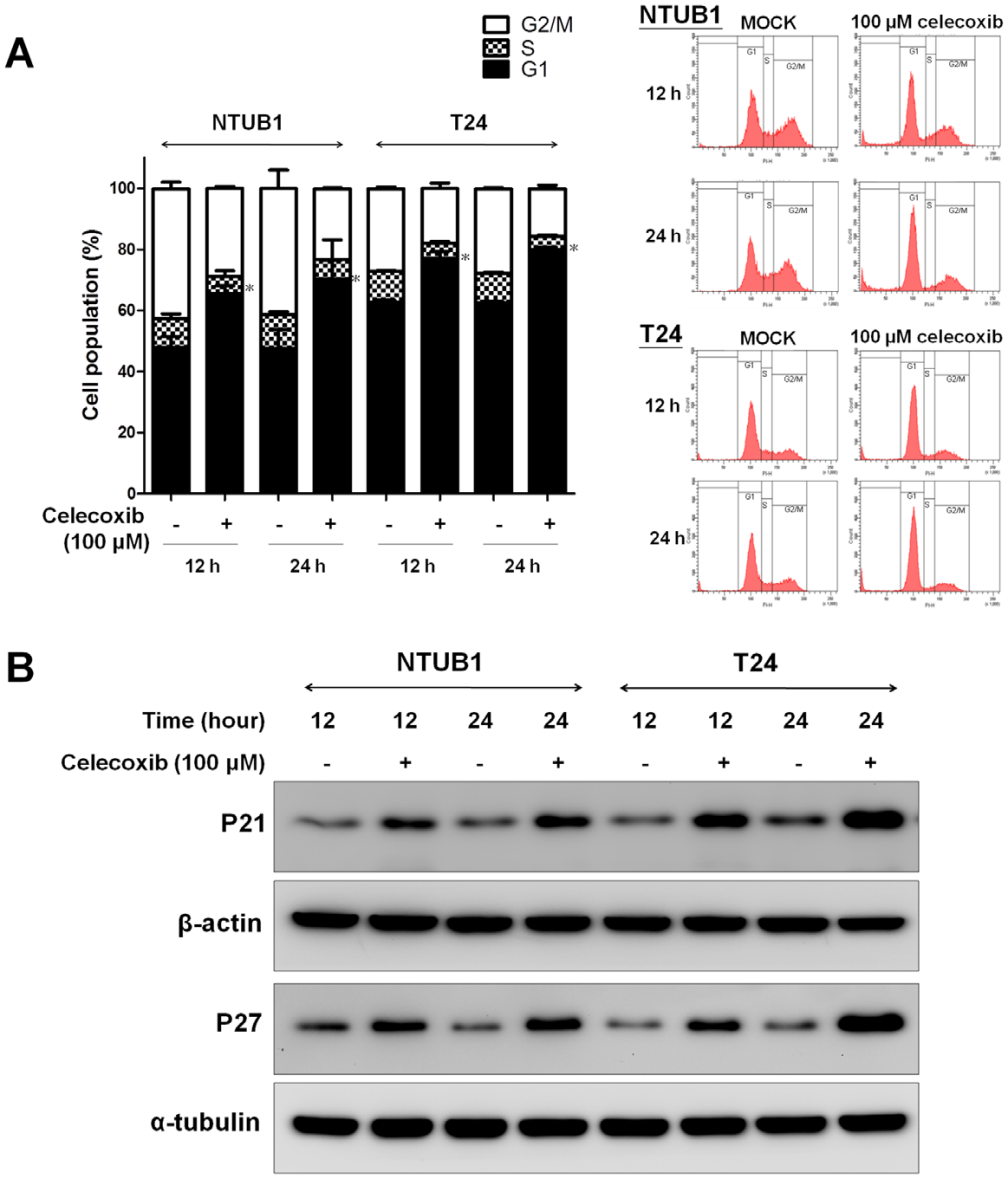
Celecoxib caused cell cycle arrest at G1 phase in NTUB1 and T24 UC cells. (A) NTUB1 and T24 cells were treated with mock (untreated) or celecoxib (100 µM) for 12 and 24 h, and cell cycle analysis was measured as described in Materials and methods. Data are presented as means ± SD of three independents experiments. * p<0.05 as compared with control. (B) The expressions of p21 and p27 at 12 and 24 h after celecoxib treatment were analyzed by Western blotting. Results shown are representative of at least three independent experiments.

Next, we determined whether celecoxib has a cell cycle arrest effect in human UC cells. Celecoxib-treated UC cells were blocked in the G1 phase after 12 and 24 h treatment (Figure 2A). Moreover, the expressions of Cdk inhibitor proteins p21 and p27 in NTUB1 and T24 cells were markedly increased at 12 and 24 h after exposure to celecoxib (100 µM) (Figure 2B).

### Celecoxib induced ER stress in human UC cells

**Figure 3:**
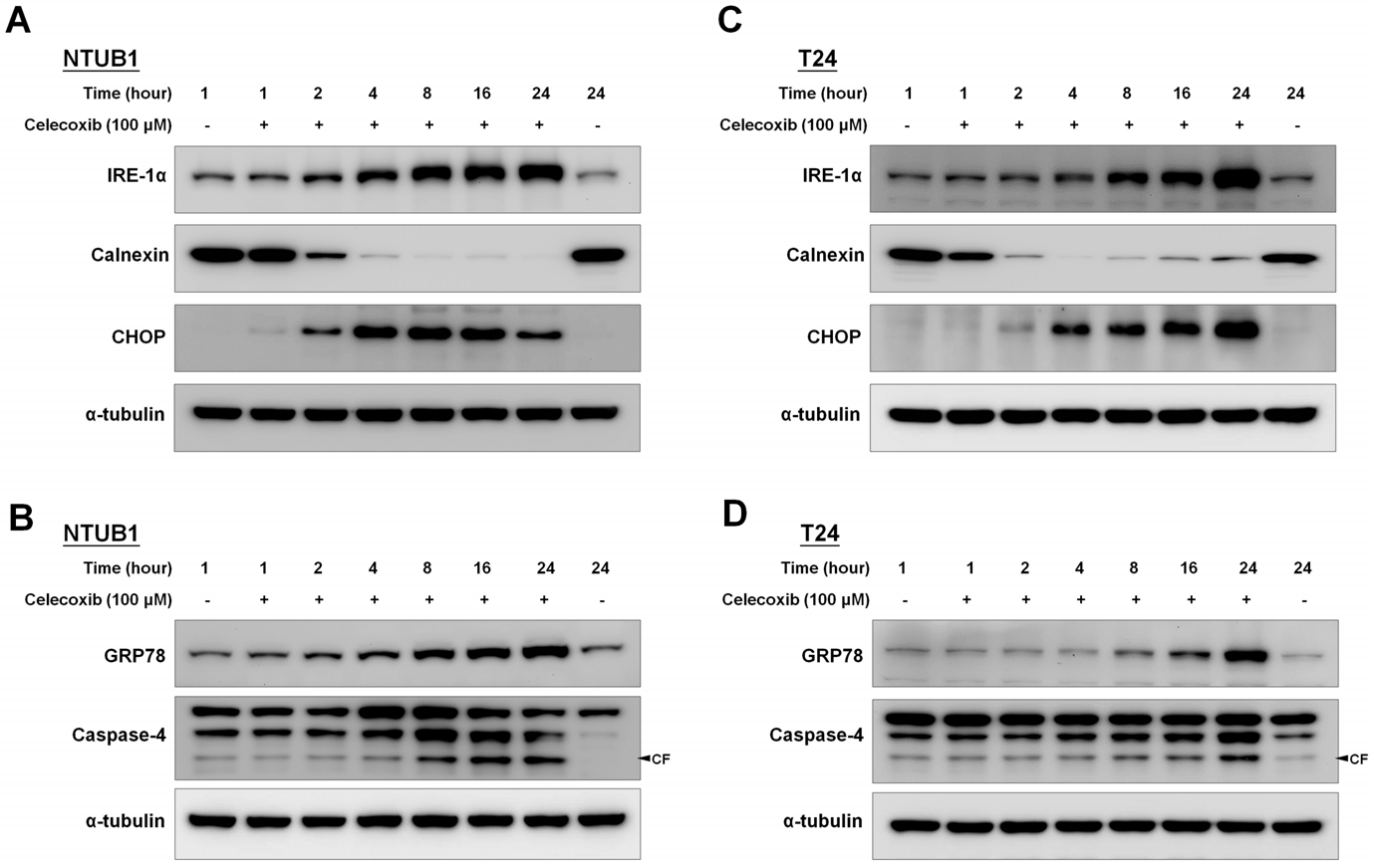
Effects of celecoxib on the ER stress-related signaling molecules IRE-1α, GRP78, CHOP, and caspase-4 in NTUB1 and T24 cells. In (A) and (B), NTUB1 cells were exposed to mock (untreated) and 100 µM celecoxib. The cell lysates were harvested at each time point and analyzed by Western blotting with specific antibodies to detect ER stress-related molecules IRE-1α, CHOP, calnexin, GRP78, and caspase-4,. CF is the abbreviation of cleaved form. (C) and (D) showed the similar experiments performed in T24 cells. Results shown are representative of at least four independent experiments.

Celecoxib has been reported to induce ER stress in several types of cancer cells [12], [17], [26]–[27]. Here, we found that treatment of NTUB1 and T24 cells with 100 µM celecoxib could also induce ER stress (Figure 3). During the 24 h exposure, celecoxib induced the protein expressions of IRE-1α, GRP78, and CHOP and the cleavage of caspase-4 in NTUB1 (Figures 3A and 3B) and T24 cells (Figures 3C and 3D). In addition, the suppression of calnexin was also shown after celecoxib (100 µM) treatment in NTUB1 (Figure 3A) and T24 cells (Figure 3C).

### GRP78 knockdown enhanced celecoxib-induced apoptosis in human UC cells

**Figure 4:**
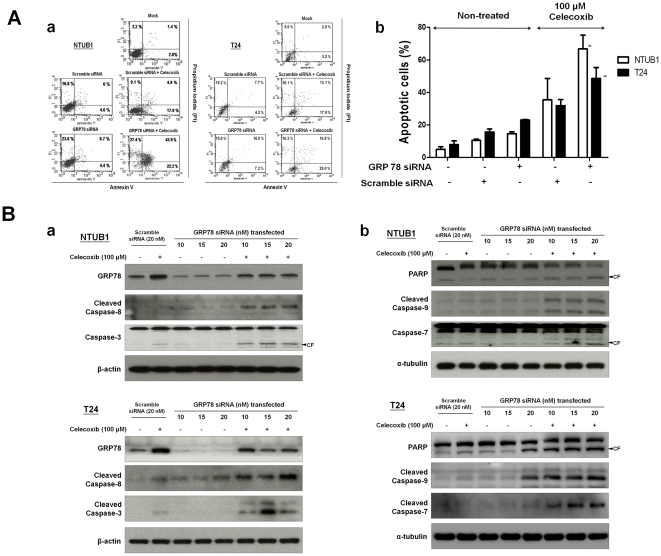
The combinative effects of celecoxib and GRP78 knockdown on apoptosis of NTUB1 and T24 cells. (A) Cells were transfected with GRP78 siRNA (10 nM) or scramble siRNA (10 nM) (as a control); then treated with 100 µM celecoxib. The combinative effect of celecoxib and GRP78 knockdown on apoptosis was determined by flow cytometry (FACS) with annexin V-FITC and PI labeling in (a). (b) Quantitative analysis of total apoptosis (early and late) population was presented. Data are presented as means ± SD of three independents experiments. * p<0.05 as compared with scramble siRNA+celecoxib. (B) Cells were transfected with 10, 15 and 20 nM GRP78 siRNA, and 20 nM scramble siRNA as control; then treated with 100 µM celecoxib. Cell lysates were harvested and analyzed by Western blotting with specific antibodies against GRP78, caspase-3, 8, cleaved caspase-3, 8 (a), caspase- 9, 7, cleaved caspase-7, 9, PARP and cleaved PARP (b). CF is the abbreviation of cleaved form. Results shown are representative of at least three independent experiments.

GRP78 has been reported to be associated with chemoresistance [28]–[29]. The celecoxib-induced expression of GRP78 raises a question concerning the relationship between GRP78 expression and apoptosis in NTUB1 and T24 cells. To clarify this issue, we used the siRNA approach to examine the role GRP78 in celecoxib-induced apoptosis in NTUB1 and T24 cells (Figure 4A). Transfection of GRP78 siRNA, which actually decreased the protein expression of GRP78 (Figure 4B), significantly enhanced the increase of cell apoptosis (Figures 4A) and the cleavage of caspases and PARP (Figures 4B) in celecoxib (100 µM)-treated NTUB1 and T24 cells. These results indicate that GRP78 expression may be correlated to the chemoresistance to celecoxib in human UC cells.

### Treatment with EGCG, a known GRP78 inhibitor, potentiated celecoxib-induced apoptosis in human UC cells

**Figure 5:**
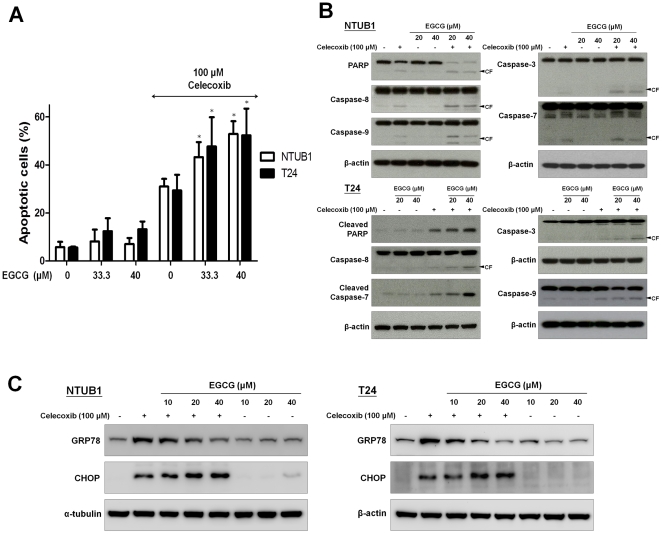
The combinative effects of celecoxib and EGCG on apoptosis in NTUB1 and T24 cells. Cells were incubated in the presence of 100 µM celecoxib and EGCG (10, 20, 33.3 and 40 µM) individually or in combination. (A) Cells were stained with annexin V-FITC and PI for apoptosis analysis by FACS flow cytometry. Results shown are representative of at least three independent experiments. Data are presented as means ± SD of three independents experiments. * p<0.05 as compared with celecoxib alone. (B) and (C) Cell lysates were harvested and analyzed by Western blotting with specific antibodies against caspase-3, 7, 8, 9, PARP, GRP78, and CHOP. CF is the abbreviation of cleaved form. Results shown are representative of at least three independent experiments.

Recently, several compounds have been discovered to be GRP78 antagonists and have anticancer activity [28]–[29]. These compounds worked in synergy with chemotherapeutic drugs to reduce tumor growth. EGCG has been reported to bind to the ATP binding domain of GRP78 and thereby blocks its function [29]–[30]. Here, we investigated the apoptosis induction effect of EGCG in combination with celecoxib on NTUB1 and T24 cells. As shown in Figure 5A, treatment with EGCG promotes celecoxib-induced apoptosis in NTUB1 and T24 cells. The combinative treatment of EGCG induced down-regulation of GRP78 and enhanced the celecoxib-induced cytotoxicity in NTUB1 and T24 cells (Figure 5B and C).

### MG132 enhanced celecoxib-induced apoptosis in human UC cells

**Figure 6:**
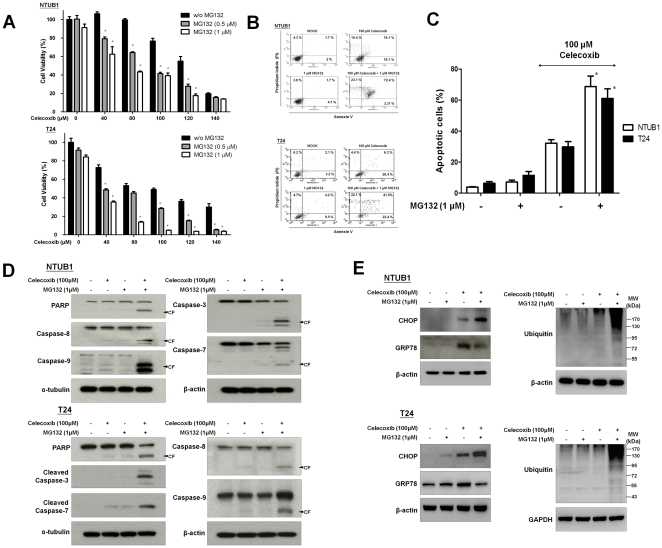
The combinative effect of celecoxib and MG132 on cell growth and apoptosis in NTUB1 and T24 cells. Cells were incubated in the presence of 100 µM celecoxib and MG132 (0.5 and 1 µM) individually or in combination. (A) Cell viability was measured by MTT assay. (B) Cells were stained with annexin V-FITC and PI for apoptosis analysis by FACS flow cytometry. (C) Quantitative analysis of total apoptosis (early and late) population was presented. (D) and (E) Cells were incubated in the presence of 100 µM celecoxib and MG132 (1 µM) individually or in combination. Cell lysates were harvested and analyzed by Western blotting with specific antibodies against, caspase-3, 7, 8, 9, PARP, CHOP, GRP78, and ubiquitin. In A and C, data are presented as means ± SD of three independents experiments. * p<0.05 as compared with celecoxib alone. In B and D, results shown are representative of at least three independent experiments.

To reduce UPR, the proteasome pathway plays a role in the degradation of unfolded protein [21], [26], [31]. It is conceivable that inhibition of proteasome may aggravate celecoxib-induced cell apoptosis due to the accumulation of unfolded protein. To test this issue, we examined the combinative effect of celecoxib and proteasome inhibitor, MG132, on NTUB1 and T24 cells. At low dose (0.5 and 1 µM), MG132 did not affect cell viability, whereas the combination of celecoxib and MG132 enhanced the cell death (Figure 6A), apoptosis (Figures 6B and 6C), and the cleavages of caspases and PARP (Figure 6D) in NTUB1 and T24 cells. Moreover, MG132 could furthermore increase celecoxib-induced ubiquitin and CHOP and downregulate GRP78 expressions in NTUB1 and T24 cells (Figure 6E). These findings also indicated that proteosome inhibitor MG132 aggravated the celecoxib-induced unfolded protein stress and potentiate the ER stress-related apoptosis.

### GRP78 knockdown sensitize LM-1685, a non-coxib COX-2 inhibitor, -induced apoptosis in human UC cells

**Figure 7:**
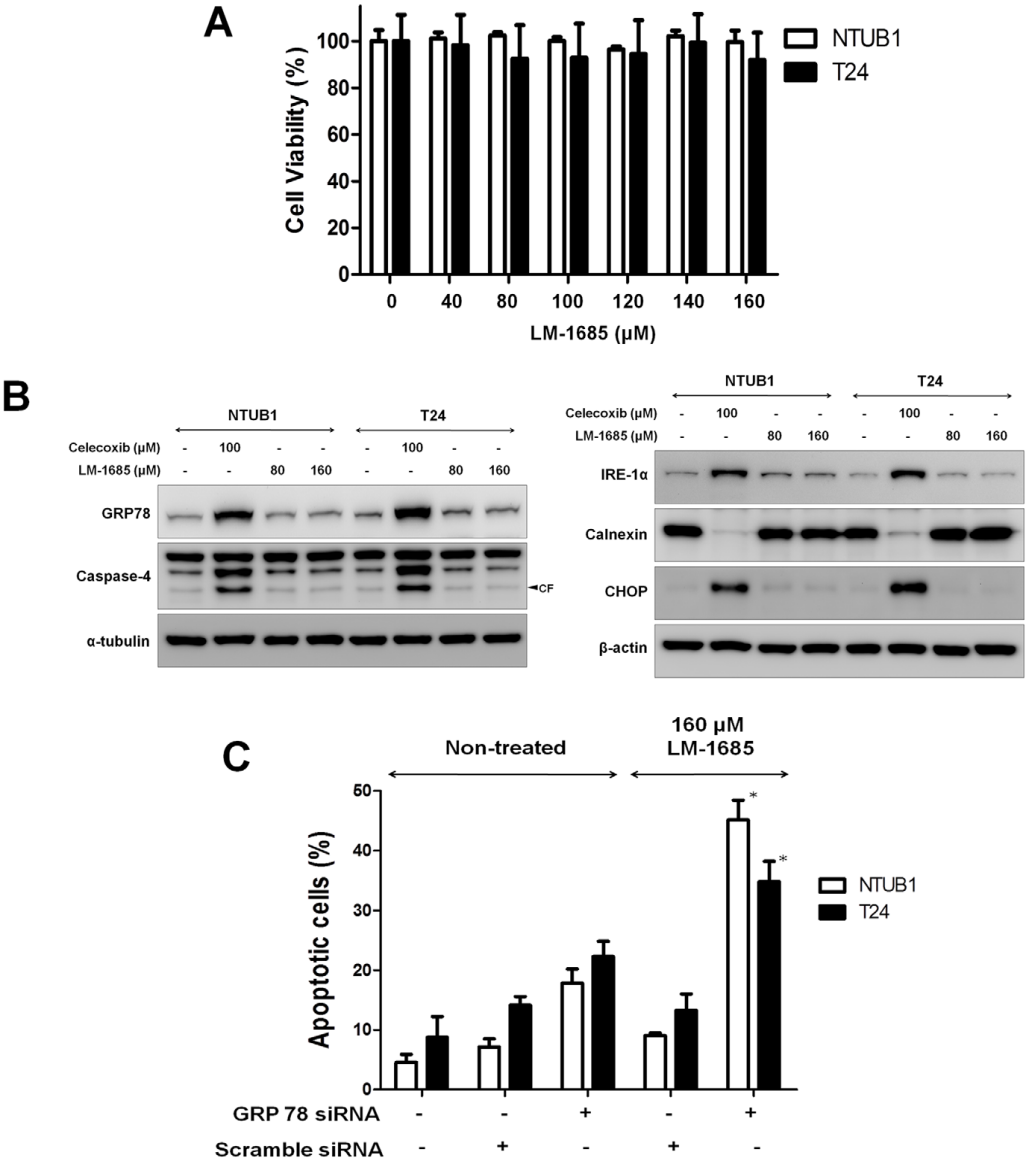
Effects of LM-1685, a non-coxib COX-2 inhibitor, on the viability and induction of ER stress-related molecules in NTUB1 and T24 cells. (A) Cells were treated with LM-1685 (0–160 µM) for 24 h. Cell viability was analyzed by MTT assay. Data are presented as means ± SD of three independents experiments. (B) Cells were treated with LM-1685 (80 and 160 µM) or celecoxib (100 µM) for 24 h. The cell lysates were harvested at each time point and analyzed by Western blotting with specific antibodies to detect ER stress-related molecules IRE-1α, CHOP, calnexin, and GRP78, and caspase-4. CF is the abbreviation of cleaved form. (C). Cells were transfected with GRP78 siRNA (10 nM) or scramble siRNA (10 nM) (as a control), and then treated with 160 µM LM-1685. The combinative effect of LM-1685 and GRP78 knockdown on apoptosis was determined by flow cytometry (FACS) with annexin V-FITC and PI labeling. The quantitative analysis of total apoptosis (early and late) population was presented. Data are presented as means ± SD of three independents experiments. * p<0.05 as compared with scramble siRNA+LM-1685.

On the contrary, celecoxib analogue LM-1685, a non-coxib COX-2 inhibitor, had no inhibitory effects on the viability of NTUB1 and T24 cells (Figure 7A). LM-1685 did not induce the expression of ER stress-related molecules after 24 h treatment (Figure 7B). Transfection with GRP78 siRNA significantly enhanced the apoptotic effect of LM-1685 in NTUB1 and T24 UC cells (Figure 7C). We believed that down-regulation of GRP78 could sensitize the drug resistance of LM-1685 to UC cells. These findings suggest the critical role of GRP78 on the survival of UC cells after COX-2 inhibitor treatment.

## Discussion

Systemic chemotherapy is the only modality to improve the survival in patients with metastatic UC. However, the treatment of metastatic UC by cytotoxic chemotherapy has reached a therapeutic plateau. To search for novel treatment modalities is imperative. COX-2 inhibitors have been studied in pre-clinical investigation as therapeutic or chemo-preventive agents in various cancers [7]–[8], [11]–[13], [15]. However, the treatment efficacy of COX-2 inhibitors in UC has not been fully explored [7]–[8], [32]–[34]. In this study, we showed that celecoxib is capable of inducing the ER stress, apoptosis, and cell death in human UC cells. GRP78 knockdown by siRNA, GRP78 inhibitor, or proteasome inhibitor effectively enhanced the celecoxib-induced caspases-regulated UC cell apoptosis.

The UPR can induce the transcription of genes encoding ER-resident chaperones to facilitate protein folding. Meanwhile, the ERAD can be activated to degrade the unfolded proteins accumulated in the ER [21]–[22]. The goal of UPR is to alleviate the cellular stress and restore proper ER homeostasis. However, if the ER stress persists intensely, these signaling pathways can trigger cell apoptosis [19]–[20]. In mammalian cells, signaling molecules PERK, IRE-1α, and ATF6 sense the presence of unfolded proteins in the ER lumen and transduce the signals to the cytoplasm and the nucleus [19], [35]. GRP78 is a main regulator of the pro-survival pathway in the UPR and plays an important role in protein folding and assembly [19], [35]. Aggregation of unfolded proteins resulted in the ER stress induction that GRP78 dissociates from the three ER transmembrane receptors (PERK, ATF-6, and IRE-1α), which leads to their activation and triggers the UPR [18], [20]. The activated PERK pathway induces downstream CHOP expression, and then triggered the cell apoptosis. Calnexin, an ER transmembrane chaperone, plays the key roles in translocation, protein folding, and quality control of newly synthesized polypeptides [18], [20].

The roles of GRP78 in tumor formation, progression and angiogenesis have been demonstrated [29], [35]. Drug resistance of cancer cells to a broad range of therapeutic agents, many of which are not directly linked to ER stress, has been attributed to GRP78. GRP78 has been shown to reduce the ER stress-related cancer cell apoptosis [28], [35]. Constitutive over-expression of GRP78 has also been reported to confer chemo-resistance in cancer therapy [28]–[29]. Down-regulation of GRP78 by siRNA or chemical inhibition has been shown to enhance the chemo-sensitivity in tumor-associated endothelial cells [29]. Recently, several compounds have been shown to be GRP78 inhibitors, which have anticancer activity and work in synergy with chemotherapeutic drugs to reduce tumor growth [28]–[30]. Chemo-resistance remains a major challenge in treatment of metastatic UC. Identifying mechanisms of drug resistance and development of new therapeutic agent are important in treatment of UC [2]. In this study, exposure of human UC cells to celecoxib actually induces UPR activation. The celecoxib-induced UPR in human UC cells is associated with the up-regulation of GRP78. GRP78 knockdown by using siRNA or chemical inhibition (EGCG) could potentiate the cytotoxic and apoptotic effect of celecoxib in UC cells. Moreover, LM1685 did not up-regulate GRP78 as celecoxib, nor did it induce cytotoxicity in human UC cells. However, GRP78 knockdown did effectively enhance celecoxib cytotoxicity and reverse resistance to LM1685. Our findings indicate the critical role of GRP78 in protecting cancer cells from COX-2 inhibitor-induced apoptosis. Down-regulation of GRP78 can significantly enhance the susceptibility to COX-2 inhibitor in UC cells.

The ubiquitin proteosome pathway is another pathway for intracellular protein degradation to maintain homeostasis during cell encounter the UPR stress [31]. A previous study has shown that a combination of celecoxib and proteosome inhibitor MG132 provides synergistic anti-proliferative effect in human liver tumor cells [21]. In the present study, we found that combined treatment with MG132 in human UC cells could potentiate celecoxib-induced cytotoxicity with concomitant down-regulation of GRP78.

Celecoxib is commonly administered orally with dosage of 200 mg twice daily, resulting in mean peak serum concentration of 1–2 µM [36]. Reported side effects of celecoxib in therapeutic dosage include cardiovascular thrombosis, congestive heart failure, gastrointestinal ulceration, renal or hepatic injury, and platelet aggregation [37]. Some reports on side effects of celecoxib in supratherapeutic dosage in clinical trial showed that there were no significant side effects in supratherapeutic dosage [38]. In our study, using in vitro methods, we chose 100 µM as the working concentration of celecoxib, a concentration much higher than the concentration corresponding to the FDA recommended maximal dose. This is in line with a variety of studies on the anti-tumor effect of celecoxib *in vitro* showing that the concentration of celecoxib needed to inhibit growth of cancer cells *in vitro* is much higher than that needed *in vivo* for bladder and other cancers [7]–[8], [10], [39]–[42]. This discrepancy indicates that tumor growth *in vivo* is determined by interactions between factors intrinsic to tumor cells and extrinsic factors such as the extracellular matrix, stromal cells, and other host factors. These extrinsic factors are generally absent under in vitro conditions. Cell culture models are often used to evaluate the therapeutic potential of COX-2 inhibitors against cancer, but it must be noted that *in vitro* results, particularly as relates to relative dose of agent used, cannot be directly extrapolated to the whole organism (*in vivo*) [42].

In conclusion, the present study showed that celecoxib can significantly inhibit the proliferation of human UC cells. The aggravated unfolded protein stress caused by down-regulation of GRP78 or by proteasome inhibitor will further enhance the celecoxib-induced UC cell apoptosis. These findings are promising and warrant further study for the development of new therapeutic strategies against UC.

## Supporting Information

Figure S1 **Both T24 and NTUB1 cells showed high level of COX-2 and GRP78 expression.**
(TIF)
