# Supplementary figures and images for: Down-Regulation of Glucose-Regulated Protein (GRP) 78 Potentiates Cytotoxic Effect of Celecoxib in Human Urothelial Carcinoma Cells
Source: PLoS One. 2012 Mar 16;7(3):e33615. doi: 10.1371/journal.pone.0033615 (PMC3306428; doi:10.1371/journal.pone.0033615)

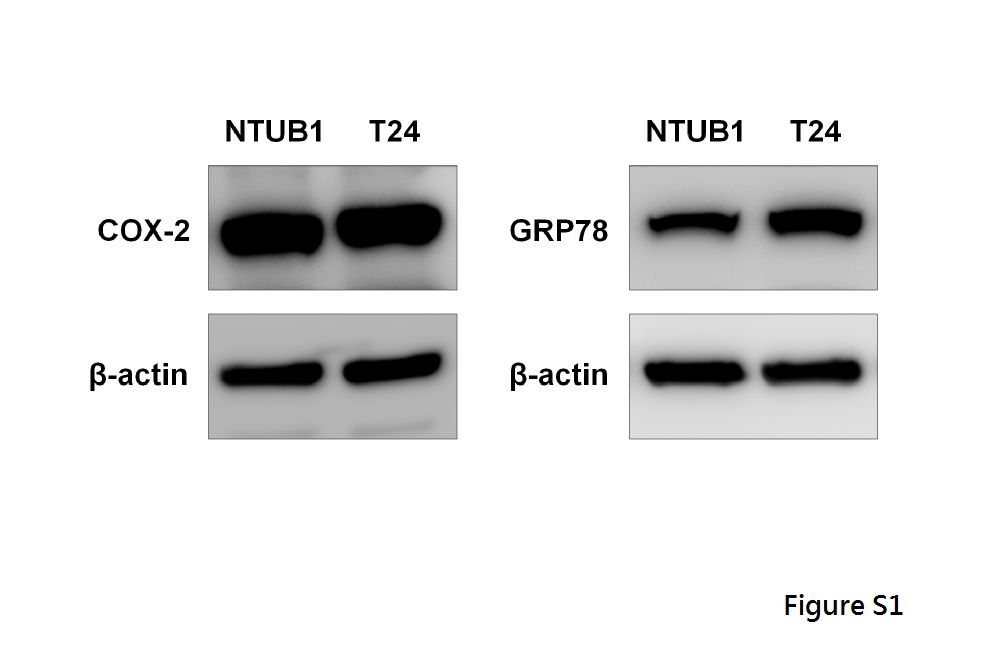

Supplement: Figure S1 — Both T24 and NTUB1 cells showed high level of COX-2 and GRP78 expression. (TIF) [file pone.0033615.s001.tif]
